# A new fabrication method for enhancing the yield of linear micromirror arrays assisted by temporary anchors

**DOI:** 10.1038/s41378-024-00679-4

**Published:** 2024-05-20

**Authors:** Xingchen Xiao, Ting Mao, Yingchao Shi, Kui Zhou, Jia Hao, Yiting Yu

**Affiliations:** 1 Ningbo Institute of Northwestern Polytechnical University, College of Mechanical Engineering, Northwestern Polytechnical University, 710072 Xi’an, China; 2 Key Laboratory of Micro/Nano Systems for Aerospace (Ministry of Education), Shaanxi Province Key Laboratory of Micro and Nanoelectromechanical Systems, Northwestern Polytechnical University, 710072 Xi’an, China

**Keywords:** Optical sensors, Micro-optics

## Abstract

As one of the most common spatial light modulators, linear micromirror arrays (MMAs) based on microelectromechanical system (MEMS) processes are currently utilized in many fields. However, two crucial challenges exist in the fabrication of such devices: the adhesion of silicon microstructures caused by anodic bonding and the destruction of the suspended silicon film due to residual stress. To solve these issues, an innovative processing method assisted by temporary anchors is presented. This approach effectively reduces the span of silicon microstructures and improves the Euler buckling limit of the silicon film. Importantly, these temporary anchors are strategically placed within the primary etching areas, enabling easy removal without additional processing steps. As a result, we successfully achieved wafer-level, high-yield manufacturing of linear MMAs with a filling factor as high as 95.1%. Demonstrating superior capabilities to those of original MMAs, our enhanced version boasts a total of 60 linear micromirror elements, each featuring a length-to-width ratio of 52.6, and the entire optical aperture measures 5 mm × 6 mm. The linear MMA exhibits an optical deflection angle of 20.4° at 110 Vdc while maintaining exceptional deflection flatness and uniformity. This study offers a viable approach for the design and fabrication of thin-film MEMS devices with high yields, and the proposed MMA is promising as a replacement for digital micromirror devices (DMDs, by *TI Corp*.) in fields such as spectral imaging and optical communication.

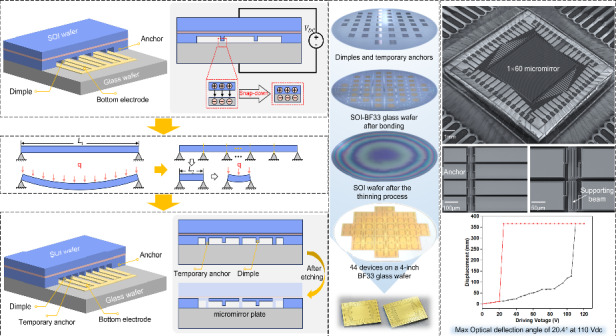

## Introduction

**Fig. 1:**
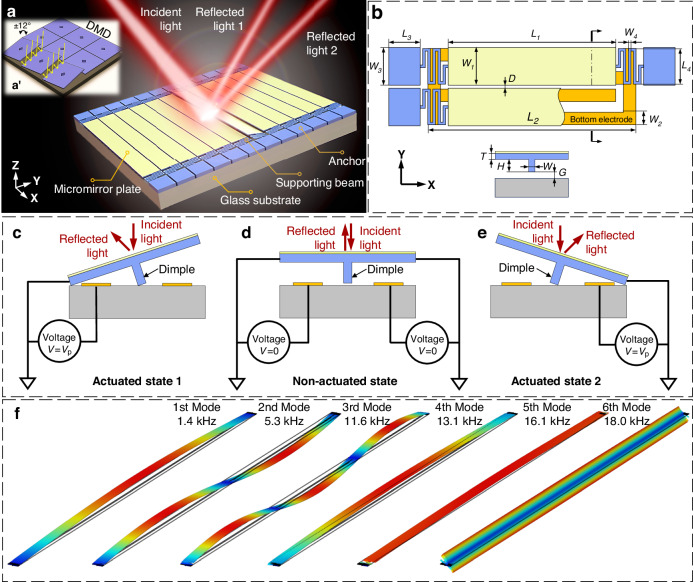
Structural design and working principle of the designed MEMS MMA. **a** Schematic diagram showing one rotational micromirror structure used to redirect the incident light (**a**’ shows the MSS of the DMD). **b** Structural parameters of a micromirror unit. **c–e** Working principle of the MMA. **f** Finite-element simulation results of modal analysis

Hyperspectral imaging (HSI) is a powerful detection tool that merges imaging and spectroscopy technologies and exhibits multiple functions across diverse domains, notably encompassing remote sensing, biomedical diagnosis, and archeology^1–3^. Across the range of demonstrated HSI techniques, the push-broom mode stands out for its unique ability to simultaneously achieve remarkable spatial and spectral resolutions. However, for such purposes, the integration of bulky moving components for line scanning is necessary, inevitably resulting in a substantial increase in overall system dimensions, weight, and power consumption. In this context, spatial light modulators (SLMs) based on the microelectromechanical system (MEMS) process have emerged as a transformative technology. These SLMs provide precise control over optical beam phases, boasting commendable attributes such as minimal energy consumption and compact form factors. They serve as cores in various optical applications, spanning from light detection and ranging (LiDAR)^4,5^, microscopic imaging^6,7^, and holographic displays^8^ to HSI^9,10^. The evolution of MEMS-based SLMs has ushered in a revolution in HSI systems. A notable example is the pixel-level programmable digital micromirror device (DMD) developed by *Texas Instruments*. This commercially representative SLM has been applied in snapshot spectral imaging systems, where it has replaced mechanical counterparts with binary-coded masks. Moreover, DMDs have been harnessed to facilitate rapid on-chip scanning in push-broom HSI systems devoid of any macro moving parts^11–13^. Nevertheless, the inherent deficiency in the structure and deflection mode of DMDs is the issue of micromirror self-shadowing (MSS) within the same row (Fig. 1a’). This phenomenon manifests as the change in illumination caused when part of the incident or reflected light is obstructed by an adjacent micromirror edge. To align with conventional optical components, indispensable adjustments require either a 45° rotation of the DMD or tilting of the whole incident light path^14–16^. Unfortunately, these modifications lead to an increased system height, thereby preventing the system’s aspiration for miniaturization.

To overcome these challenges, a novel linear micromirror array (MMA) was previously implemented by our group, where each micromirror was rotated around its long axis, resulting in a large length-to-width ratio^17^. This device was manufactured using the bulky silicon micromachining process. Linear micromirrors without release holes could significantly reduce the loss of optical energy, thereby improving the overall performance of HSI systems. The key to the successful fabrication of the MMA lies in the layer transfer process based on anodic bonding, which involves transferring a suspended monocrystalline silicon thin film from a silicon-on-insulator (SOI) wafer to a BF33 glass wafer containing addressing electrodes. However, two primary challenges emerge during the layer transfer process, which severely reduces device yield. First, a substantial processing voltage, typically on the order of several hundred to 1000 V, must be applied between the silicon and BF33 glass to establish reliable bonding. This process is susceptible to a well-known phenomenon referred to as “snap-down”, where suspended diaphragms are stuck to the glass substrate after the anodic bonding process^18^, ultimately leading to the “self-locking” of micromirror structures. Second, during the thinning process of an SOI wafer, a large-area, freely suspended silicon film can buckle and rupture due to residual compressive stress in the buried oxide (BOX) layer.

A variety of innovative approaches have been explored to solve these complex issues effectively. In the case of snap-down damage, one strategy involved increasing the distance between the glass substrate and silicon microstructures to reduce electrostatic forces. However, this method may not be suitable for structures such as parallel-plate micromirror units, as a narrow gap between capacitive plates is typically preferred to optimize the driving force. Alternatively, maintaining an electric potential of zero between suspended silicon microstructures and bottom electrodes on the glass during the anodic bonding process can prevent damage to the suspended microstructures^19^. Regarding the issue of cracking and buckling deformation in silicon films, one approach is to employ zero-stress materials, such as single-crystalline silicon wafers. Nonetheless, a micromirror thickness of <10 μm employing this substrate proves to be exceedingly challenging, primarily because of the absence of an etching stop layer. Another method involves mitigating the influence of residual stress without altering the fundamental fabrication process or materials by increasing the thickness of the structural membrane to resist deformation. While this approach reduces the silicon film deformation, it also induces higher driving voltages for devices.

This paper presents a novel temporary-anchor-assisted fabrication method for enhancing the yield of linear MMAs. The core concept underlying this method involves reducing the span of suspended microstructures by strategically placing multiple temporary anchors in noncritical structural areas, such as the gaps between adjacent micromirror units and other regions that are slated for eventual etching. This strategy substantially bolsters the robustness of these structures. Of key significance is our successful realization of wafer-scale, high-yield manufacturing of MMA. This MMA comprises linear micromirror units featuring a length-to-width ratio exceeding 50, and it includes a submicron gap between the silicon micromirror structure and the glass substrate. Furthermore, our research contributes novel manufacturing methodologies tailored for ultrathin and miniaturized devices. The developed MMA serves as a pivotal spatial light modulator for diverse potential applications, encompassing his microscopic imaging, and LIDAR, among other techniques.

## Results and discussion

### Design of the temporary-anchor-assisted micromirror array

The proposed linear MMA is presented in Fig. 1a. This novel MMA design features individually addressable micromirror elements. Within each element, the micromirror plate is suspended above a shared bottom electrode by securely anchored serpentine supporting beams. A dimple structure enhances the robustness and mitigates pull-in failure^20^. Compared with DMDs, the MSS in the same row is eliminated by utilizing a linear micromirror structure. Comprehensive specifications for the designed MMA are provided in Fig. 1b. Figure 1c–e elucidates its operational principle, with a focus on a single micromirror unit for clarity. Upon the application of a pull-in voltage (*V*_p_) from the left (as depicted in Fig. 1c), the micromirror rotates to the left. A similar scenario occurs when the driving voltage is applied from the right (as illustrated in Fig. 1e)^21,22^. This working principle underscores the pivotal role of the gap between the dimple and the glass substrate in the process of micromirror movement. Any adhesion of the dimple to the glass substrate obstructs the normal micromirror deflection.

For the entire MMA, a simple driving strategy is designed in which micromirror plates are independently arranged, and the driving voltage is applied in turn, with all left bottom electrodes grounded and a similar grounding approach to all right bottom electrodes, as depicted in Fig. 1b. This configuration is well suited for the on-chip scanning required by the push-broom HSI. The scanning process involved sequential rotation of 60 micromirror units in a uniform direction, either to the left or right. Notably, only one micromirror unit is activated at each address (see Fig. 1a), channeling optical information to the subsequent spectral dispersion subsystem. Each micromirror unit must return to its original position before the next unit is addressed. The rapid response characteristic of electrostatically driven structures, in conjunction with the driving circuit, ensures that all the micromirror units scan one by one.

Furthermore, modal analysis of a micromirror unit is conducted, and the simulation results are presented in Fig. 1f. The resonant frequencies for the first to sixth modes are 1.4, 5.3, 11.6, 13.1, 16.1, and 18 kHz, respectively. The crucial parameter values for the proposed MEMS-based MMA are clearly detailed in Table 1. In contrast to the linear MMA utilized in our prior research^17^, the current version introduces a micromirror with an elevated length-to-width ratio to realize the finer resolution of spatial light modulation.

**Table 1:** Device parameters of the designed MMA

Parameter	Values (μm)
Micromirror plate	*L*_1_: 5000, *W*_1_: 95, *D*: 5, *T*: 5.5
Bottom electrode	*L*_2_: 5180, *W*_2_: 40
Anchor	*L*_3_: 150, *W*_3_: 93
Supporting beam	*L*_4_: 95, *W*_4_: 5
Dimple	*H*: 8.9, *W*: 5, *G*: 0.6

**Fig. 2:**
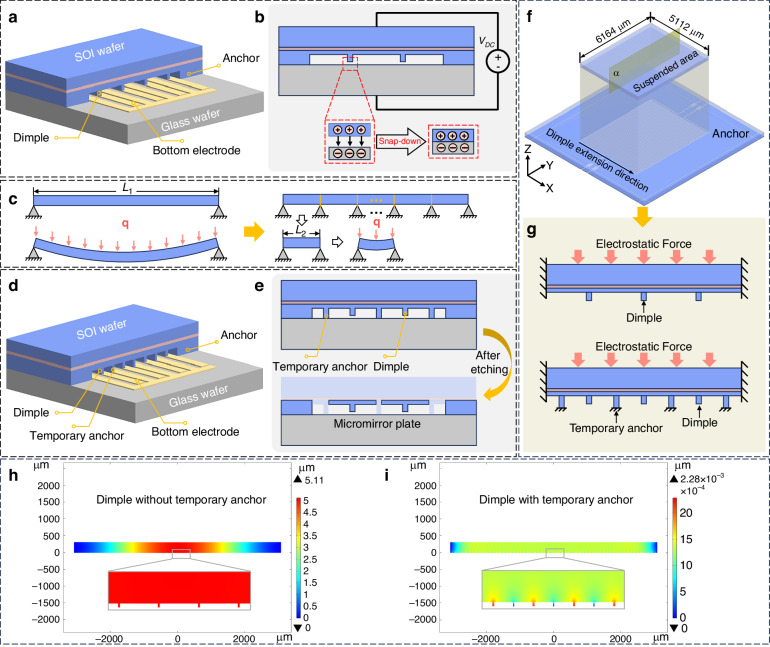
Schematic diagram of temporary bonding anchors. **a** 3D diagram of a wafer bonding structure with only dimple structures. **b** Wafer bonding cross-section with only dimple structures and a snap-down phenomenon that occurred during the bonding process. **c** Deformation of a simply supported beam with and without multiple fulcrums. **d** 3D diagram of the wafer bonding structure with temporary anchors. **e** Wafer bonding cross-section with temporary anchors and discrete micromirror plates formed after etching temporary anchors. **f**, **g** Simulation models for analyzing the influence of temporary anchors. **h**, **i** Simulated deformation during bonding without and with temporary anchors

As mentioned above, the layer transfer process is a crucial step for the successful fabrication of our linear MMA. This process includes transferring a monocrystalline silicon thin film from an SOI wafer to a BF33 glass wafer and relies on anodic bonding. In Fig. 2a, a three-dimensional (3D) diagram illustrates the anodic bonding structure without temporary anchors. The figure shows the suspended silicon microstructures, including dimples, with a small gap positioned above the glass wafer. However, as shown in Fig. 2b, the significant electric potential drop and the relatively narrow gap between the two substrates can generate a strong electrostatic force during the anodic bonding process. This force strives to bring the dimples into contact with the glass wafer, leading to permanent adhesion.

In engineering, a span is the distance between two intermediate supports, such as a beam or bridge. The span length is a significant factor in explaining the strength and size of a beam because it determines the maximum bending moment and deflection. According to the mechanics of the materials, the maximum bending moment is *M*_max;_ thus, the generated deflection *δ*_max_ in Fig. 2c can be defined as

1 $${M}_{\max }=\frac{q{L}^{2}}{8}$$

2 $${\delta }_{\max }=\frac{5{M}_{\max }{L}^{2}}{48EI}=\frac{5q{L}^{4}}{384EI}$$

where *q*, *L*, *E*, and *I* are the uniformly distributed load, length of the beam between two supports (i.e., the span length), modulus of elasticity, and area moment of inertia, respectively. Equation (2) shows that under the condition of a constant uniformly distributed load, if the span length is doubled, the maximum bending moment will quadruple, and the deflection will increase by a factor of sixteen. For this reason, as shown in Fig. 2c, the span length is reduced from L_1_ to L_2_ by adding multiple fixed supports, and the deformation of the beam is, therefore, greatly suppressed.

Inspired by these phenomena, a temporary-anchor-assisted processing method is developed. A schematic diagram is shown in Fig. 2d, where multiple temporary anchors are periodically distributed between two adjacent dimples to effectively diminish the span of the suspended silicon microstructure while enhancing its rigidity. As shown in Fig. 2e, due to the height difference between the temporary anchors and dimples, when the temporary anchors are in contact with the glass substrate, a submicron gap forms between the dimples and the substrate. A remarkable quality of this method is that the temporary anchor located at the gap of the MMA does not require any additional processing. When the micromirror structure is released, it can be etched away, which will not affect the integrity or optimal performance of the linear MMA (see Fig. 2e). In the designed MMA, the entire area measures 6164 μm × 5112 μm, and the material is suspended above the glass substrate. A central section parallel to the long-axis direction is selected, which has the largest span. A finite element simulation model was established to analyze the deformation of the suspended silicon microstructure under the influence of the bonding voltage (Fig. 2f, g). The maximum deformation can reach 5.11 μm without temporary anchors, which greatly exceeds the gap between the dimples and the glass substrate. In contrast, the maximum deformation caused by the addition of temporary anchors is only 2.28 × 10^−3^ μm (Fig. 2h, i). This result clearly underscores the key role of temporary anchors in significantly reducing the deformation of the suspended silicon microstructure and ensuring the presence of submicron gaps between the dimples and the glass substrate.

### Experimental results

**Fig. 3:**
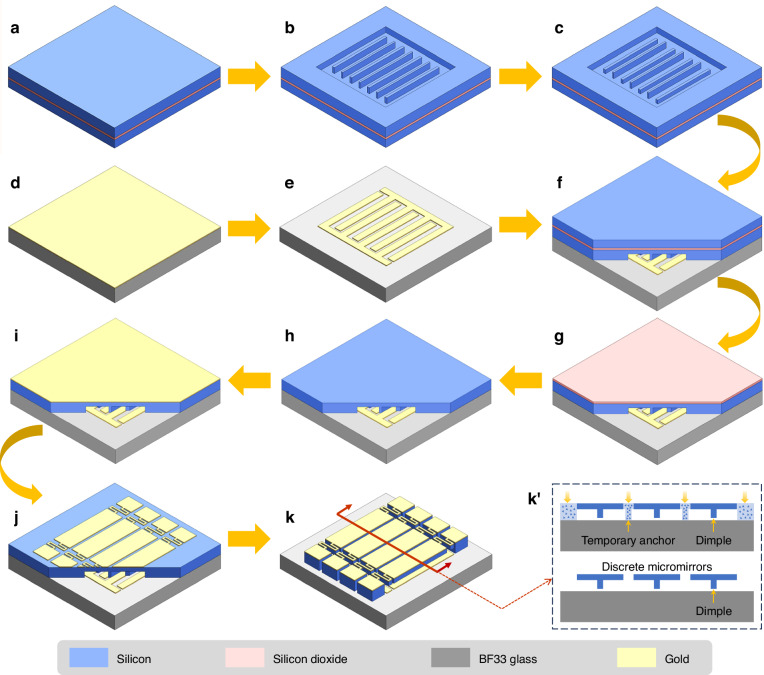
Fabrication process of the MMA with temporary anchors. **a** Start with an SOI wafer. **b** Fabrication of dimples, temporary anchors, and anchor structures. **c** The difference in height between dimples and temporary anchors created by etching. **d** BF33 glass as the bottom electrode with an Au film on top. **e** Define the common ground electrode and electrical pads. **f** Anodic bonding of SOI and BF33 glass substrates. **g** DRIE of the SOI handle layer. **h** Removal of SOI buried oxide (BOX). **i** Deposition of the Au film as the reflective surface. **j** Patterning of the Au film. **k** Release of micromirror plates and removal of temporary anchors (as shown in **k**’, enlarged illustrative view)

The temporary-anchor-assisted linear MMA was fabricated using a four-mask bulk micromachining process, with monocrystalline silicon serving as the structural material. A detailed flowchart of the fabrication process is shown in Fig. 3. Initiating the process included precleaning a 4-inch SOI wafer in a piranha solution to eliminate any organic contaminants (Fig. 3a). This SOI wafer comprised distinctive layers including a 15-µm-thick device layer (n-type, 0.01–0.001 Ω cm), a 2-µm-thick BOX layer, and a 350-µm-thick handle layer. Subsequently, the device layer was patterned using standard photolithography techniques to create dimples and temporary anchors, followed by dry etching via the deep reactive ion etching (DRIE) process (Fig. 3b). To precisely define the height difference between the dimples and temporary anchors, an additional dry etching step was applied (Fig. 3c). A thorough depiction of this fabrication step is available in Supplement 1, Fig. S1. Concurrently, a 4-inch BF33 glass wafer was coated with a 200-nm-thick Au film utilizing Cr as the adhesion layer (Fig. 3d). The abovementioned Au film was then patterned through wet etching, facilitating the formation of the bottom common ground electrode and electrical pads (Fig. 3e). The integration of the BF33 glass and SOI at the wafer level was completed through the anodic bonding technique (350 °C, 1000 V) (Fig. 3f). Subsequent steps included removal of the handle layer utilizing the DRIE technique with the BOX layer serving as an effective etch stop (Fig. 3g), followed by elimination of the BOX layer (Fig. 3h). To mitigate undesirable warping and breakage of the silicon film on the device layer, the subsequent fabrication utilized deliberately designed air channels to ensure pressure balance. Additionally, an annealing step was implemented after bonding to enhance the micromirror reflectivity. This step involved depositing a 200-nm-thick gold film and selectively patterning it using wet etching (Fig. 3i and j, respectively). Ultimately, both the micromirror plates and serpentine supporting beams were precisely defined and released via the DRIE process (Fig. 3k). Notably, the strategic placement of temporary anchors within the gaps between adjacent micromirrors was a critical innovation of the process. This technique ensured that once discrete micromirrors were formed, the temporary anchors beneath them were exposed to the etching environment, thus extending the process time to guarantee the complete removal of these sacrificial structures (Fig. 3k’). A high filling factor of 95.1% was achieved and demonstrates the efficacy of this process.

**Fig. 4:**
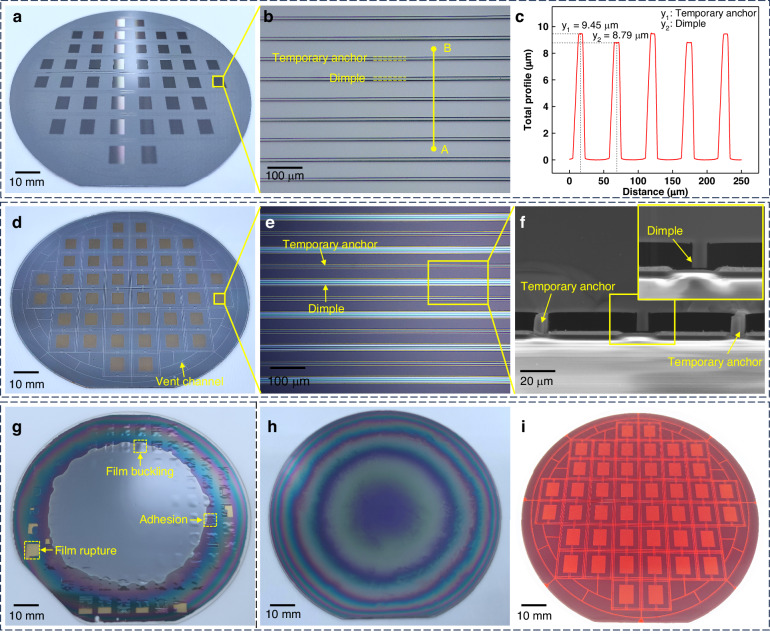
Results of wafer bonding based on temporary anchors. **a**, **b** Optical images of dimples and temporary anchors. **c** Measured height difference between dimples and temporary anchors. **d**, **e** Optical images of the SOI-BF33 glass wafer after bonding, showing the details of the dimples and temporary anchors. **f** SEM image of the bonding section including both dimples and temporary anchors. **g** Optical image of an SOI wafer after the thinning process without temporary anchors, showing severe membrane buckling and cracking. **h** Optical image of an SOI wafer after the thinning process with temporary anchors, showing a flat and entire membrane. **i** The whole silicon structure illuminated from the back

Figure 4a depicts the wafer after the preparation of the dimples and temporary anchors, and Fig. 4b provides a more detailed view of these structures, revealing their periodic arrangement in strip-like configurations. Precise measurements using a DektakXT stylus profilometer confirmed a height difference of 660 nm, as shown in Fig. 4c. The outcome after anodic bonding is displayed in Fig. 4d, where the dark gray region signifies the bonded area, indicative of impeccable bonding quality. Figure 4e further shows a microscopy image, with temporary anchors depicted in dark gray and dimples in blue-white due to the existing gap in the glass substrate. The cross-sectional scanning electron microscopy (SEM) image in Fig. 4f provides a clear view of the gap between the dimples and the glass substrate. To assess the results after handle layer thinning with and without temporary anchor structures, we conducted comparative experiments. Figure 4g shows such a wafer without temporary anchor structures, corresponding to the processing step outlined in Fig. 3g. Nonuniform etching led to the complete removal of the handle layer at the edge of the wafer, exposing the underlying oxide layer. However, the silicon film had already exhibited buckling and cracking before the etching process was completed.

When fabricating the continuous silicon film as described above, the residual tensile stress inside ensures the flatness of the membrane due to its doubly fixed-end boundary. In contrast, if the residual compressive stress exceeds the Euler buckling limit, the membrane buckles. The Euler buckling limit of a doubly fixed beam is given by^23^

3 $${\sigma }_{{\rm{Euler}}}=\frac{-{\pi }^{2}}{3}\frac{E{H}^{2}}{{L}^{2}}$$

where *H* is the thickness of the beam. According to Eq. (3), the buckling of a film can be avoided if the total residual stress is in tension or compression and weaker than the limit. The temporary-anchor-assisted processing technique can enhance the Euler buckling limit by decreasing the span *L* of the suspended silicon film, thereby improving the yield of the SOI wafer thinning process. As depicted in Fig. 4h, the handle layer of the SOI wafer was entirely eliminated, resulting in a smooth and flawless surface on the remaining device layer. The resulting enhancement in surface quality undeniably facilitates the following processing stages. Figure 4i shows an optical image of the completed thin silicon film illuminated from below with white light. The film exhibits a red hue when exposed to a white light source, indirectly affirming its thin and uniform nature. Additionally, ventilated grooves crisscrossed between structures. The introduction of grooves on the device layer surface served dual purposes: preventing air entrapment within the SOI wafer chamber during bonding and enhancing the overall bonding quality. During the SOI substrate thinning process, connecting the chamber on the SOI wafer’s device layer to the DRIE reaction chamber ensured balanced pressure on both sides of the SOI wafer, effectively preventing rupture of the device layer.

**Fig. 5:**
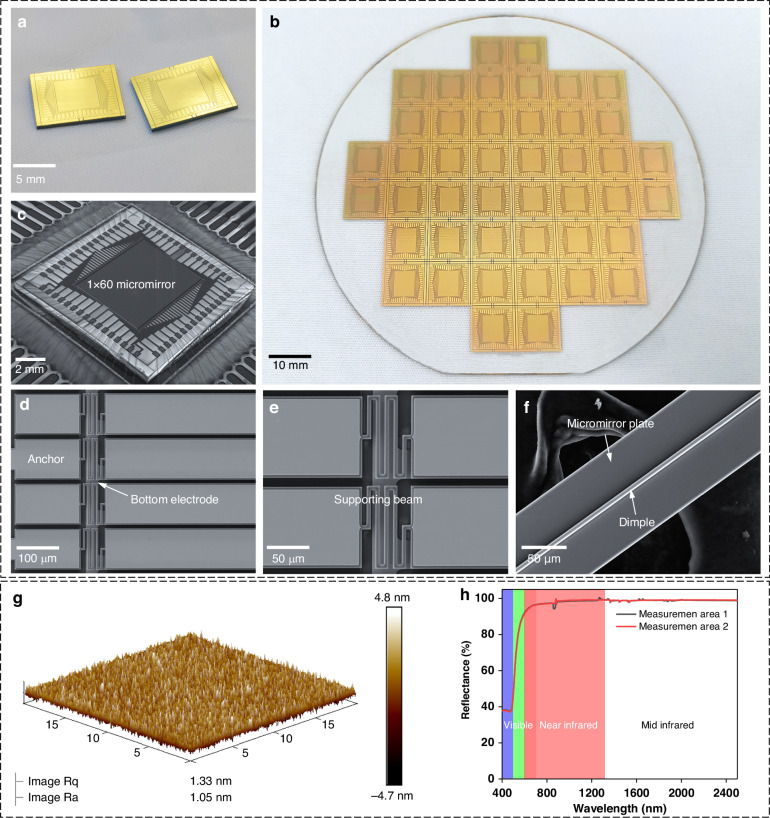
Fabricated MEMS linear MMAs. **a** Photograph of two MMAs. **b** A total of 44 MMAs were integrated on a 4-inch BF33 glass wafer. **c** SEM image of an intact MMA. **d** Close-up view of anchors and the bottom common ground electrode. **e** Close-up view of the serpentine supporting beams. **f** Close-up view of a micromirror plate with a dimple structure. **g** Surface roughness of the deposited reflective gold layer as measured by AFM. **h** Refractive index of the micromirror surface as a function of wavelength

Figure 5a displays a photograph of two 10 mm × 10 mm linear MMA chips, each featuring an optical aperture of more than 5 mm × 6 mm. In Fig. 5b, there are a total of 44 devices on a 4-inch BF33 glass substrate, and each device contains unblemished structures, highlighting the advantages of the novel fabrication method proposed in this paper and greatly enhancing the yield of our linear MMAs. Figure 5c shows an SEM image of a complete MMA. A high filling factor of 95.1% is obtained by using a parallel-plate electrostatic driving strategy. Additionally, detailed views of several key structures, including anchors, common bottom electrodes, support beams, dimples, and micromirror surfaces, are given (as shown in Fig. 5d–f). Subsequently, we performed a meticulous surface quality assessment using atomic force microscopy (AFM), revealing a surface roughness Ra of 1.05 nm in height variation across the 20 μm × 20 μm unit area of the gold-coated surface (Fig. 5g). In this study, we applied a mirror surface comprising 10 nm chromium and a 200 nm metallic layer. Figure 5h graphically depicts the micromirror reflectance data acquired through a microarea spectrophotometer. The linear MMA represents an example of exceptional optical efficiency, with a specular reflectance exceeding 93% within the wavelength range of 560–2500 nm. Specular reflectance measurements were precisely performed using a spectrophotometer (PerkinElmer Lambda 650). The radius of curvature (ROC) of a micromirror is the factor that determines its flatness. A large ROC value indicates that the micromirror has a flatter surface. To assess the ROC of our developed micromirror, we employed a Veeco/Wyko NT1100 3D optical profiler. A three-dimensional image of the micromirror surface is shown in Supplement 1, Fig. S2a, with the dashed line indicating the length direction. Over a span of 300 μm along the length of the micromirror unit, the surface profile undergoes an approximate change of 30 nm. Simultaneously, measurements at two distinct positions revealed ROC values of 21.81 and 16.34 cm (Supplement 1, Fig. S2b), confirming the outstanding flatness of the micromirror along its length direction.

**Fig. 6:**
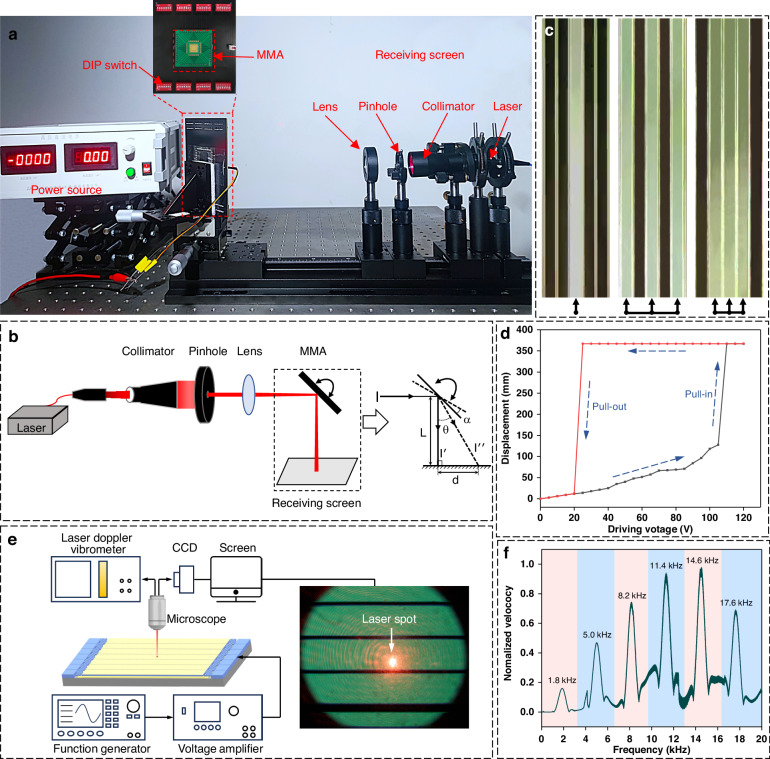
Experimental tests of the developed MMA. **a** Experimental setup. **b** Schematic diagram for calculating the rotational angle. **c** Some typical working states for the MMA, with one micromirror, two micromirrors, and three alternative micromirrors actuated. **d** Operational behavior of the micromirror unit when actuated by an increased or decreased driving voltage, showing a clear hysteresis loop. **e** Block diagram for measuring the resonant frequencies of micromirrors by LDV. **f** Normalized velocity of a micromirror as a function of its excitation frequency from 1 kHz to 20 kHz

The fabricated MMA chip was wire-bonded to a printed circuit board (PCB) for comprehensive electrical and optical testing. To determine the maximum optical rotational angle of the MMA, we devised a fundamental optical path system, as depicted in Fig. 6a. A laser beam with a wavelength of 640 nm was employed and was directed onto the micromirror surface through a sequence of optical components, including a beam expander, aperture, and lens. Subsequently, the laser beam was reflected onto the receiving screen. As the micromirror was driven, the optical rotational angle was obtained by following the geometrical principle elucidated in Fig. 6b. *L* was 988.6 mm, and *d* was 366.9 mm, resulting in an optical rotational angle *θ* of 20.4° and thus a mechanical rotational angle *α* of 10.2°. Figure 6c shows the microscopy results obtained with varying numbers of micromirrors. At a driving voltage of 0 V, the reflection image was dark. As the driving voltage increases, the reflection image progressively brightens, indicating increased light reflection into the objective lens. These experimental findings confirm the feasibility of the proposed MMA chip for future HSI systems, where the slit width can be adjusted by altering the number of columns per modulation unit. This versatility enables diverse scanning modes, including coarse scanning, fine scanning, and regional scanning, akin to our prior HSI systems^11^. Furthermore, we evaluated the electromechanical performance of the proposed device using the same setup. A direct current (DC) voltage source capable of generating high voltages was employed. When DC voltage was applied to the micromirror unit, we determined its rotational state by recording the spot position on the receiving screen. By applying a series of driving voltages, we established the relationship between the spot displacement and the driving voltage, as demonstrated in Fig. 6d. Our testing results revealed a pull-in voltage of ~110 V. Additionally, the curve of electromechanical performance exhibited the tilt angle/voltage hysteresis phenomenon commonly observed in electrostatic parallel-plate actuators. Figure 6e is the block diagram of the measurement setup, utilizing a microscopic laser Doppler vibrometer (LV-S01-M, Soptop Inc.). Figure 6f shows the resonant mode responses of the fabricated micromirror unit. The first six resonant frequencies of the micromirror are derived, and the sixth order is 17.6 kHz. The resonant frequency provided by the finite-element simulation was 18 kHz for the 6th mode. As explained above, differences between the measured and simulated values can be attributed to fabrication variations. We also measured the switching response time of the micromirror by applying a 110 V step voltage. As shown in Supplement 1 and Fig. S3a and b, when we define the switching time of the micromirror as the time required for the micromirror to move and settle to the maximum displacement of the micromirror, the micromirror has a switching time of 0.51 ms (Supplement 1, Fig. S3a). Moreover, it takes 0.36 ms for the micromirror to be switched off (Supplement 1, Fig S3b). The observed discrepancy in the durations of activation and deactivation likely stems from the inherent differences in the capacitor charging and discharging times.

**Fig. 7:**
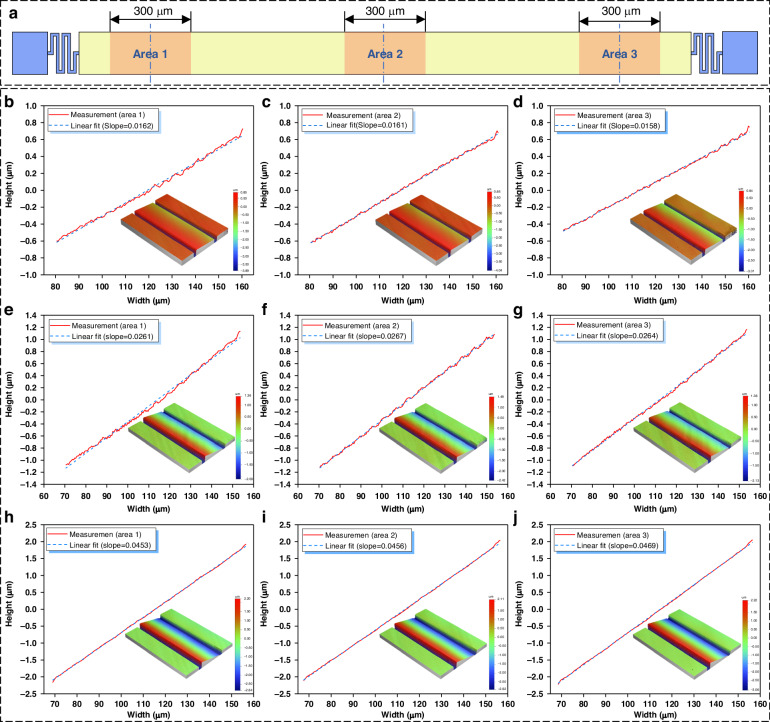
Surface flatness and deflection uniformity of the deflected micromirror. **a** Distribution of the three measurement areas on the micromirror. **b–d** Micromirror profiles at a driving voltage of 43 V. **e–g** Micromirror profiles at a driving voltage of 63 V. **h–j** Micromirror profiles at a driving voltage of 93 V

As shown in Fig. 7a, we divided an individually driven micromirror into three 300 μm-long regions (along its length axis) to evaluate the surface flatness and deflection uniformity when actuated. A Veeco/Wyko NT1100 optical profiler was used to construct surface profiles under different driving voltages (43, 63, and 93 V). Deflection data from the central axis of each region were selected and linearly fitted to quantitatively characterize the deflection angles of the micromirrors. Figure 7b–d depicts the deflection profiles of the three regions under a 43 V driving voltage, with a standard deviation of deflection angles of 0.0097 across these regions. Figure 7e–g and h–j correspond to driving voltages of 63 and 93 V, with standard deviations of 0.0140 and 0.0378, respectively. All these results demonstrate excellent deflection flatness and uniformity when the micromirror units are actuated, ensuring excellent optical performance when the method is applied for HSI.

## Conclusion

In this study, we address two critical issues encountered in the fabrication process of MEMS-based MMAs. Namely, we overcome bonding adhesion and silicon film rupture by designing a novel temporary-anchor-assisted fabrication approach. Through the strategic use of periodically arranged temporary anchors, the structural span is successfully reduced, thereby improving the force distribution and further enhancing the load-bearing capability. Consequently, this method enabled wafer-level, high-yield fabrication of MMAs. The resulting devices exhibit flat and smooth surfaces, achieving a 95.1% filling factor through 60 linear micromirror structures. Extensive testing demonstrated that when driven by a 110 V bias voltage, the maximum optically reflected angle of the developed micromirror was 20.4°. In summary, this approach provides novel insights for the design and fabrication of thin-film MEMS devices while introducing an innovative approach to optimize the processing of existing devices.

### Supplementary information

Supplement 1
