# Supplementary material for: A new fabrication method for enhancing the yield of linear micromirror arrays assisted by temporary anchors
Source: Microsyst Nanoeng. 2024 May 20;10:63. doi: 10.1038/s41378-024-00679-4 (PMC11102899; doi:10.1038/s41378-024-00679-4)
Supplement: Supplementary file 1 — Supplement 1 [file 41378_2024_679_MOESM1_ESM.docx]

**Supplementary information**

**A new fabrication method for enhancing the yield of linear micromirror arrays assisted by temporary anchors**

Xingchen Xiao, ^1,2^ Ting Mao, ^1,2^ Yingchao Shi, ^1,2^ Kui Zhou, ^1,2^ Jia Hao, ^1,2^ and Yiting Yu^1,2,3,*^

*1 Ningbo Institute of Northwestern Polytechnical University, College of Mechanical Engineering, Northwestern Polytechnical University, Xi’an 710072, China.*

*2 Key Laboratory of Micro/Nano Systems for Aerospace (Ministry of Education), Shaanxi Province Key Laboratory of Micro and Nano Electro-Mechanical Systems, Northwestern Polytechnical University, Xi’an 710072, China.*

* [yyt@nwpu.edu.cn](mailto:yyt@nwpu.edu.cn)

**Supplementary Materials:**

Figs. S1-S3

**The formation of height difference between dimples and temporary anchors**

Fig. S1 presents a comprehensive fabrication process flow, with a focus on illustrating the formation of height difference between dimples and temporary anchors. Fig. S1a depicts a three-dimensional view for a better understanding. To accurately represent the height variation between the dimples and temporary anchors, we have chosen a longitudinal cross-sectional view. As depicted in Fig. S1b, following the initial etching of the device layer, the dimples are of the same height as the temporary anchors. The next step involves spin-coating and patterning photoresist to create a mask for the secondary etching process, as shown in Fig. S1c. Then the deep reactive ion etching method is used for the second etching of the temporary anchors, achieving a precise sub-micron-level height discrepancy between temporary anchors and dimples, as illustrated in Fig. S1d. Finally, we remove the photoresist from the surface of the SOI wafer using an oxygen plasma etching technique.


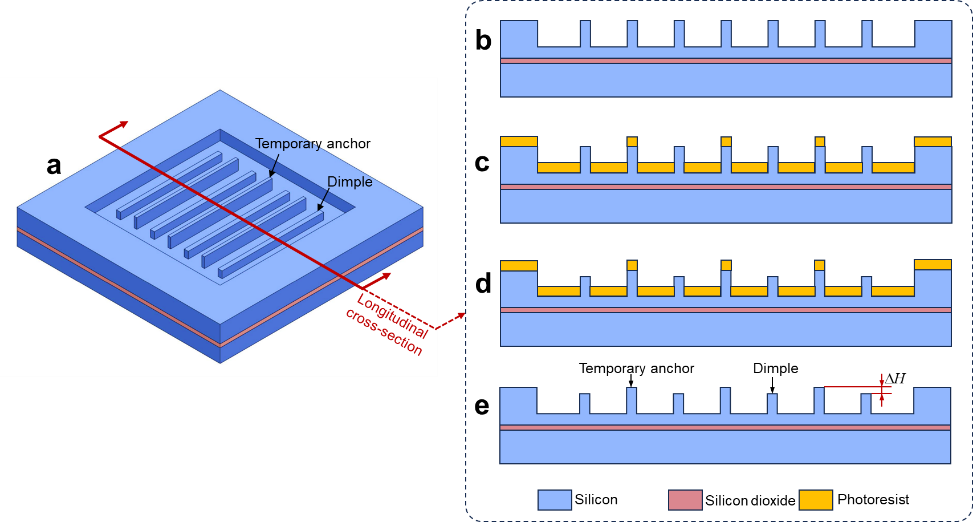


**Fig. S1 Fabrication process of the height difference between temporary anchors and dimples.** **a** Three-dimensional diagram of dimples and the temporary anchors. **b** Fabrication of dimples, temporary anchors and anchor structures. **c** Spin-coating and patterning the photoresist. **d** Etching temporary anchors. e Removing the photoresist.

**Radius of curvature (ROC) of micromirror**


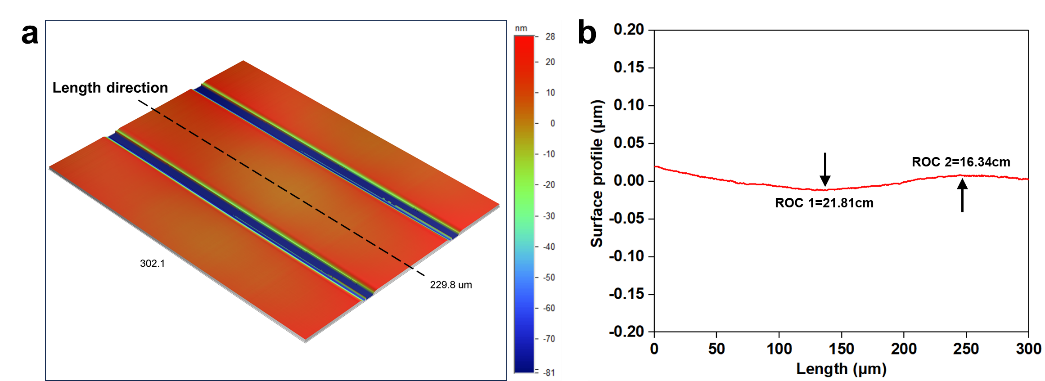


**Fig. S2 Measured radius of curvature (ROC) of the fabricated micromirror using a 3D surface profiler.** **a** 3D measurement result. **b** Measured ROCs along the micromirror’s length direction.

**Switching response time of micromirror**


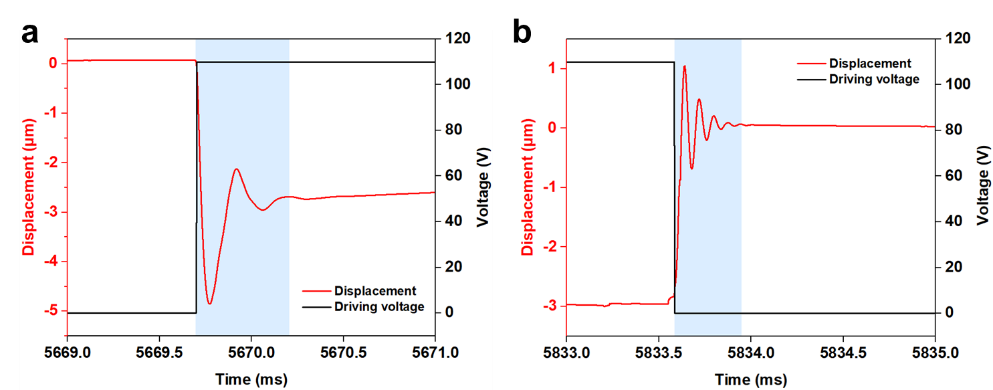


**Fig. S3 Transient responses of the micromirror.** **a** turn-on responding time. **b** turn-off responding time.
